# A novel morphological assessment approach for abdominal aortic aneurysms: automated segmentation and measurement using a semi-supervised deep learning algorithm

**DOI:** 10.3389/fcvm.2026.1956738

**Published:** 2026-09-30

**Authors:** Guangkuo Wang, Jingyi Zeng, Min Li, Fang Li, Shangqian Li, Zhigang Zhang

**Affiliations:** 1 Department of Cardiovascular Surgery, Jiangmen Central Hospital, Jiangmen, Guangdong, China; 2 The First School of Clinical Medicine, Guangdong Medical University, Zhanjiang, Guangdong, China

**Keywords:** abdominal aortic aneurysm, deep learning, image segmentation, morphological measurement, semi-supervised learning

## Abstract

**Background:**

Morphological assessment of abdominal aortic aneurysm (AAA) is crucial for preoperative strategy formulation, but traditional manual assessment is time-consuming and subjective.

**Objectives:**

This study aims to develop a novel semi-supervised deep learning (DL) framework for the automatic and accurate segmentation and measurement of AAA.

**Methods:**

In this study, a semi-supervised learning algorithm was developed based on computed tomography angiography scans collected retrospectively from 88 AAA patients. The algorithm employed 16 labeled cases for supervised training and incorporated 47 unlabeled cases through consistency regularization to achieve accurate segmentation of the AAA lumen, aneurysm neck, and iliac arteries. To evaluate the model's segmentation performance, the Dice similarity coefficient (DSC) and 95% Hausdorff distance (HD95) were utilized, and the agreement between automatically derived measurements and manual annotations was assessed.

**Results:**

Among the models evaluated, Swin-UNETR achieved the best segmentation performance with 97.3% accuracy. The DSC was 0.965 ± 0.025 and 0.931 ± 0.021, and HD95 was 2.01 ± 0.39 mm and 2.93 ± 0.28 mm in the internal and independent test sets, respectively. Automation and expert measurements showed generally good-to-excellent agreement based on intraclass correlation coefficient (ICC) analysis. In addition, average time per case decreased from 53.08 ± 8.17 to 4.18 ± 0.90 min, mouse movement distance from 91.62 ± 9.95 to 13.03 ± 3.12 m, and clicks from 672.62 ± 129.47 to 157.69 ± 0.90 times (all *P* < 0.001).

**Conclusions:**

The proposed Swin-UNETR algorithm enables fast, reliable, and reproducible automated segmentation and measurement of key anatomical details of AAA.

## Introduction

Abdominal aortic aneurysm (AAA) is characterized by an irreversible, localized dilation of the abdominal aorta, which may progress to rupture and pose a life-threatening risk if not timely diagnosed and managed (1–4). Notably, the surgical risk associated with AAA is closely tied to the morphological features of the lesion site (5–8). Accurate evaluation of key morphological paramm relies heavily on precise segmentation of computed tomography angiography (CTA) images. However, the manual or semi-automatic segmentation methods currently employed in clinical practice are associated with several limitations, including time intensity, substantial subjectivity, and a high dependence on operator expertise, which significantly hinder their efficiency and reproducibility in routine clinical applications (9–11).

To address these challenges, deep learning (DL)-based automatic segmentation approaches have demonstrated considerable potential, among which convolutional neural network architectures such as U-Net have emerged as particularly effective (12, 13). Nevertheless, these fully supervised models generally require large volumes of high-quality, pixel-level annotated data for training. The high cost and prolonged effort involved in acquiring such labeled datasets have become critical challenges limiting the widespread adoption of DL techniques in medical imaging (14).

In this context, semi-supervised learning (SSL) offers a promising solution. This method can jointly use a small amount of labeled data and a large amount of unlabeled data for model training, and significantly reduce the dependence on labeled data while ensuring performance (15). In recent years, SSL algorithms based on consistent regularization and pseudo-label mechanisms, especially the technical route of integrating data enhancement and contrast learning strategies, have made significant progress in improving the ability of models to learn effective features from unlabeled data, providing a solid theoretical basis for the application of SSL in medical image analysis (16, 17). Inspired by this, this study aims to construct a Swin-UNETR algorithm that integrates automatic segmentation and measurement to achieve rapid and accurate AAA assessment.

## Methods

### Study design and data preprocessing

In this retrospective study, abdominal CTA scans from 88 patients with AAA were collected from Jiangmen Central Hospital in Guangdong Province, China, between January 2020 and September 2025. All patients were scanned using a dual-source computed tomography (CT) system (Siemens Healthcare, Forchheim, Germany). The scanning protocol included a collimator width of 128 × 0.6 mm. Axial images were reconstructed at the optimal systolic phase with a slice thickness of 0.75 mm. The study was approved by the institutional review board of the hospital, and the requirement for informed patient consent was waived.

All data were randomly partitioned into three independent subsets: a) The training set (*n* = 63, including 16 labeled cases and 47 unlabeled cases) was used for model parameter estimation and iterative weight optimization; b) The internal validation set (*n* = 10) served to tune hyperparamm, monitor training convergence, and guide model selection; c) The independent test set (15 cases) served as an independent dataset that was not involved in the training process, enabling an unbiased assessment of the final model's segmentation accuracy and measurement consistency.

To enhance the model's generalization capability and robustness, a systematic data preprocessing and augmentation pipeline was applied to all CTA scans prior to model training. The specific procedure was as follows: (a) At the intensity level, the original CTA intensities (in Hounsfield units, HU) were uniformly truncated to the range of −200 HU to 600 HU, corresponding to a window width of 800 HU and a window level of 200 HU. The processed intensities were subsequently linearly normalized to the interval [0,1]. This step effectively mitigated intensity variations arising from differences in scanning equipment and contrast agent administration protocols; (b) At the spatial level, voxel resampling was employed to standardize all images to an isotropic resolution of 1.0 mm^3^. Furthermore, automated cropping and alignment were performed based on the region of interest (ROI) to preserve critical anatomical structures of the abdominal aorta, including its major branch vessels, while minimizing interference from surrounding organs.

### AAA image annotation

Voxel-based segmentation of the key anatomical structures of AAA, including the aneurysm sac, neck, and iliac arteries, was independently performed by two radiologists with over five years of experience in abdominal vascular imaging. The annotation process strictly adhered to the double-blind principle: without access to the algorithm's prediction results or each other's labeling information, both senior observers conducted meticulous, slice-by-slice delineation using the medical image processing software MIMICS (Materialise HQ, Belgium).

In order to ensure the quality of annotation, this study introduced a dispute arbitration mechanism: if there were significant differences between the segmentation results of the two senior observers (such as unclear anatomical boundaries), the third senior observer with more than 10 years of experience would review and make a final decision to establish the final “gold standard” mask. Furthermore, to assess the reliability and robustness of the labeled data, interobserver agreement between the two senior observers was quantitatively evaluated.

### SSL process and settings

The SSL training framework proposed in this study comprises two complementary branches: supervised and unsupervised learning. This approach leverages a limited amount of labeled data alongside a large volume of unlabeled data through collaborative training. The detailed workflow is illustrated in Figures 1A,B. In the supervised branch, weak augmentation strategies are applied to preprocess the small set of labeled data (18). Subsequently, a segmentation loss function is employed to guide the model in learning the anatomical features of the AAA from the labeled data, thereby ensuring accurate model-based segmentation performance.

**Figure 1:**
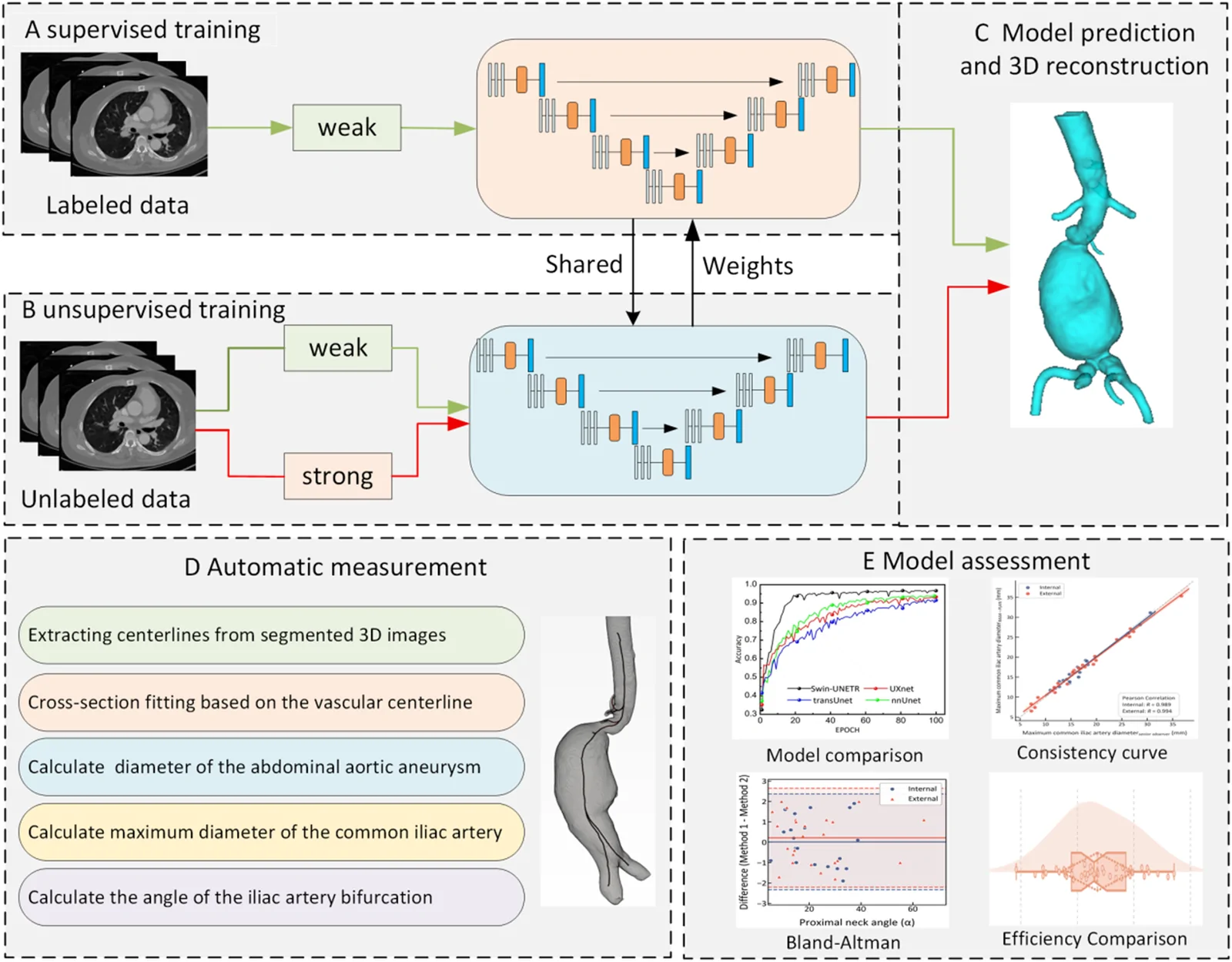
Overall framework. **(A)** Supervised training; **(B)** Unsupervised training; **(C)** Model prediction and 3D reconstruction; **(D)** Automatic measurement; **(E)** Model evaluation.

The model of this study is based on PyTorch framework (version 1.12.1, Meta AI, Menlo Park,CA,USA) and MONAI framework (version 1.0.1, MONAI Consortium). The workstation equipped with 13th Gen Intel Core i9-13900K CPU, 32 GB memory and NVIDIA RTX 4080 (24 GB memory) GPU ensures high efficiency and reliability of model training and verification.

During model training, the AdamW optimizer was employed to optimize the overall loss function, with an initial learning rate of 1 × 10^−4^ and a weight decay coefficient of 1 × 10^−5^ to mitigate overfitting. The learning rate was adjusted using a cosine annealing schedule over a period of 20 epochs, gradually reducing to a minimum value of 1 × 10^−6^ to ensure stable convergence in the later stages of training. The entire training process spanned 100 epochs with a batch size of 2. To compensate for the small batch size, a gradient accumulation strategy was adopted, accumulating gradients over 4 steps before each parameter update.

### Backbone network architecture

To establish the optimal segmentation strategy for the complex anatomical structure of AAA, this study designs a comprehensive comparative experimental framework encompassing mainstream medical image segmentation architectures. Three representative network models currently prominent in the field are selected as research subjects: nnU-Net, based on a pure convolutional neural network (CNN); TransUNet, which integrates CNN with the Transformer mechanism; and UXNet, leveraging a large-kernel convolution design. Through systematic comparative analysis, Swin-UNETR is ultimately chosen as the core backbone network for subsequent in-depth investigation and structural optimization. This network combines the strengths of CNNs in local feature extraction with the Transformer's capability to model long-range dependencies. By employing a hierarchical window-based self-attention mechanism with a 7 × 7 × 7 window size, it captures the long-range spatial relationships of vascular structures while ensuring segmentation continuity. The U-Net encoder-decoder architecture, together with skip connections, enables multi-scale feature fusion. As a result, the model achieves precise aneurysm neck delineation and preserves critical anatomical details, while effectively suppressing noise interference—making it well-suited to meet the high demands for segmentation accuracy in AAA analysis.

### SSL consistency regularization strategy

To address the challenge of limited labeled data and enhance model generalization, this study proposes a consistency regularization-based semi-supervised learning strategy. Specifically, both weak and strong augmentation operations are applied to the same input sample. The weak augmentation, aligned with the supervised branch, introduces only minimal geometric perturbations to preserve essential structural information. In contrast, a composite perturbation approach—incorporating elastic deformation (deformation coefficient: 0.05–0.1), Gaussian noise (variance: 0.01–0.02), and random contrast adjustment (contrast coefficient: 0.8–1.2)—is employed in the strong augmentation to simulate diverse variations commonly encountered in clinical images. The augmented pairs are fed into a Swin-UNETR architecture with shared weights, yielding corresponding predictions from the weakly augmented $\left(\;p^{\omega }\right)$ and strongly augmented $\left(\;p^{s}\right)$ branches. A consistency constraint is then established by minimizing the discrepancy between these two predictions, encouraging the model to learn anatomical features of AAA that are robust to input perturbations.

### Loss function design

The overall training objective was formulated as:

$$L_{\mathrm{total}}=L_{\sup}+L_{\mathrm{con}}$$

where $L_{\sup}$ represents the supervised segmentation loss calculated from labeled cases and consists of the Dice loss and cross-entropy loss:

$$L_{\sup}=L_{\mathrm{Dice}}+L_{\mathrm{CE}}$$

The weights of Dice loss and cross-entropy loss were both set to 1.0. $L_{\mathrm{con}}$ represents the voxel-level mean squared error consistency loss between the predictions generated from weakly and strongly augmented versions of the same unlabeled images. The consistency weight *μ* was gradually increased from 0 to 1.0 during the first 20 training epochs and remained constant thereafter.

In order to use a small amount of labeled data and a large amount of unlabeled data at the same time, the total loss of this study is defined as:

$$L=L_{\sup}+\mu L_{\mathrm{cons}}$$

Here, μ represents the weight of the unsupervised loss.

Supervised loss $L_{\sup}$: The segmentation loss is calculated on the predicted results of the labeled data and the true value labels. In order to alleviate the class imbalance, Dice and cross-entropy are used:

$$L_{\sup}=\lambda_{1}L_{\mathrm{dice}}+\lambda_{2}L_{\mathrm{ce}}$$

Consistency loss $L_{\mathrm{cons}}$: weak enhancement and strong enhancement were applied to the unlabeled data, respectively, and the prediction $p^{\omega }$ and $p^{s}$ were obtained by input the shared weight NETwork, and the consistency constraint was realized by minimizing the difference between the two.

$$L_{\mathrm{cons}}=\|\;p^{\omega }-p^{s}{\|}_{2}^{2}$$

### Automated geometric measurement development

In order to realize the automatic extraction of the key clinical paramm of AAA and replace the traditional manual measurement method, this study introduces a morphological measurement module based on skeletonization after the segmentation model (Figure 1D). Through three steps of center line extraction, orthogonal section fitting and parameter calculation, the clinical geometric paramm of the lesion were accurately obtained. Details of the steps are as follows:

1. *Center line extraction.* For the 3D binary mask generated by the segmentation model, a morphological opening operation with a 3 × 3 × 3 structuring element was applied to eliminate small noise artifacts. Subsequently, the Zhang-Suen thinning algorithm was employed to extract the central skeleton lines of the abdominal aorta and bilateral iliac arteries. To mitigate potential jagged irregularities arising during the thinning process, cubic spline interpolation was performed to smooth the extracted skeleton lines. This ensured that the centerlines accurately reflected the true anatomical orientation of the vessels, thereby providing a reliable foundation for subsequent quantitative analysis.
2. *Orthogonal section fitting.* Along the smoothed centerline, normal planes perpendicular to the local tangent vector were sequentially constructed at 1 mm intervals. Vessel cross-sections were obtained by computing the intersection between these planes and the vessel boundary. This approach guarantees that each cross-section represents the maximal diameter plane of the vessel segment, effectively minimizing measurement errors caused by angular deviations or oblique slicing.
3. *Parameter calculation.* For standardized morphological assessment, the aneurysm neck was defined as the non-aneurysmal infrarenal aortic segment extending from immediately below the lowest renal artery to the beginning of the aneurysm sac. The vascular centerline was defined as the central trajectory through the segmented lumen and was used to derive local vessel axes and orthogonal measurement planes. For the continuous cross sections obtained through curve fitting, the following paramm were automatically computed: (a) The maximum diameter of aneurysm: all cross sections were traversed, the minimum circumscribed circle method was used to calculate the equivalent diameter of each section, and the maximum diameter of the aneurysm area and its corresponding spatial position were located and output; (b) The maximum diameter of the iliac artery: the minimum circumscribed circle method was used to calculate the equivalent diameter by traversing the cross section of the left and right iliac artery segments, and the maximum diameter and its corresponding spatial position of the bilateral iliac arteries were output; (c) Aneurysm neck diameter: the equivalent diameter of each cross section in the defined interval of proximal aneurysm neck was calculated by the minimum circumscribed circle method, and the representative value of the interval was used to output the aneurysm neck diameter; (d) Length of aneurysm neck: the centerline distance from immediately below the lowest renal artery to the beginning of the aneurysm sac was calculated and reported as the aneurysm neck length; (e) Proximal neck angle: Based on the local tangent vector of the central skeleton line between the neck segment and the tumor segment, the Angle between the two main directions was calculated by the dot product of the vector and output; (f) Aneurysm *β* angle: the angle between the longitudinal axis of the aneurysm neck and the longitudinal axis of the aneurysm sac was calculated from the corresponding centerline-derived directional vectors; (g) Iliac artery bifurcation angle: the angle between the centerline-derived directional vectors of the left and right iliac arteries at the aortic bifurcation was calculated and reported as the iliac bifurcation angle.

### Evaluation of model

To comprehensively assess model performance, this study designed evaluation metrics based on two dimensions: segmentation accuracy and clinical consistency, using manual segmentations and parameter measurements performed by senior observers as the reference standard (Figure 1E).

With respect to segmentation performance, the Dice Similarity Coefficient (DSC) and the 95% Hausdorff Distance (HD95) were employed to evaluate the quality of segmentation. Let the predicted segmentation be denoted as *P* and the reference standard as G; then

$$DSC=\frac{2|P\cap G|}{\left|p|+|G\right|}$$

DSC values range from [0,1], with closer to 1 indicating a higher degree of overlap.

HD95 was used to measure segmentation boundary errors and reduce extreme outlier effects. Let the set of boundary points be ∂P and ∂G, respectively, and the point to set distance be $d(x,S)=\mathrm{mi}n_{y\varepsilon S}\|x-y\|$, then

$$HD95(P,G)=per({\left\{d(\;p,\partial G)\right\}}_{\;p\in \partial P}\cup {\left\{d(g,\partial P)\right\}}_{g\in \partial G})$$

The intraclass correlation coefficient (ICC) and Bland-Altman analysis were employed to assess the consistency between the key anatomical paramm of AAA measured by the algorithm and those obtained via manual measurement. Meanwhile, the total operation time, number of mouse clicks, and distance of mouse movement were compared between the algorithm's automatic analysis and the manual procedures performed by senior observers.

### Statistical analysis

The normality of distributions was evaluated using the Kolmogorov–Smirnov test. Quantitative data that followed a normal distribution were summarized as mean ± standard deviation, and group comparisons were conducted using Student's *t*-test. For quantitative data that did not follow a normal distribution, results were reported as median and interquartile range, and group comparisons were performed using the Mann–Whitney *U* test. Count data were expressed as *n* (%), and categorical variables were compared using the chi-square test and Fisher's exact test, as appropriate. For the comparison of model performance, the Wilcoxon signed-rank test was employed. A two-sided *P*-value < 0.05 was considered statistically significant. All statistical analyses were carried out using SPSS software, version 26.0 (SPSS, Inc., Armonk, New York).

## Results

### Baseline characteristics

Preoperative CTA scan data from 88 patients with AAA were ultimately included in this study. All patients were randomly assigned to either thedevelopment cohort [*n* = 73, including the training set [*n* = 63] and internal validation set [*n* = 10]] or the independent test set (*n* = 15, 17.05%). The median age of the patient population was 69.0 years (IQR, 65.0–73.25 years), and 90.91% (80 patients) were male. Except for the prevalence of atherosclerosis (*P* < 0.001), there were no significant differences in other baseline paramm between the two cohorts (*P* > 0.05). Detailed baseline characteristics of the patients are presented in Table 1.

**Table 1:** Baseline characteristics of the study population.

Characteristic	Overall (*n* = 88)	Development cohort (*n* = 73)	Independent test set (*n* = 15)	*P* value
Age, years	69.00 (65.00, 73.25)	69.00 (65.00, 73.00)	71.00 (66.50, 75.00)	0.494
Male, *n*(%)	80 (90.91)	66 (90.41)	14 (93.33)	1.000
SBP, mmHg	128.00 (117.00, 141.25)	128.00 (113.00, 140.00)	132.00 (122.00, 146.50)	0.091
DBP, mmHg	77.00 (68.00, 85.00)	76.00 (68.00, 84.00)	79.00 (73.50, 86.00)	0.230
SOAT, days	12.50 (1.00, 60.00)	10.00 (1.00, 60.00)	14.00 (3.50, 45.00)	0.617
Atherosclerosis, *n*(%)	11 (12.50)	1 (1.37)	10 (66.67)	<.001
Marfan syndrome, *n*(%)	21 (23.86)	17 (23.29)	4 (26.67)	1.000
Inflammatory diseases, *n*(%)	1 (1.14)	0 (0.00)	1 (6.67)	0.170
Diabetes mellitus, *n*(%)	17 (19.32)	14 (19.18)	3 (20.00)	1.000
Hypertension, *n*(%)	56 (63.64)	47 (64.38)	9 (60.00)	0.748
Hyperlipidemia, *n*(%)	2 (2.27)	1 (1.37)	1 (6.67)	0.313
Coronary heart disease, *n*(%)	8 (9.09)	6 (8.22)	2 (13.33)	0.893
Atrial fibrillation, *n`*(%)	1 (1.14)	0 (0.00)	1 (6.67)	0.170
Stroke, *n*(%)	1 (1.14)	0 (0.00)	1 (6.67)	0.170
COPD, *n*(%)	12 (13.64)	8 (10.96)	4 (26.67)	0.230
Smoking, *n*(%)	72 (81.82)	59 (80.82)	13 (86.67)	0.867
Alcohol consumption, *n*(%)	15 (17.05)	13 (17.81)	2 (13.33)	0.966

SBP, Systolic blood pressure; DBP, Diastolic blood pressure; SOAT, Symptom onset to hospital admission time; COPD, Chronic obstructive pulmonary disease.

### Model training and architecture selection

Figure 2A presents the performance curve of different models, including Swin-UNETR, TransUNet, UXNet, and nnUNet. As shown in the performance curve on the left, the Swin-UNETR model adopted in this study exhibits the fastest reduction in loss during the early training phase (the first 20 epochs), indicating superior learning efficiency for AAA image features. In the later stage of training (beyond 60 epochs), the loss of the Swin-UNETR model stabilizes and reaches the lowest value among all models, demonstrating its excellent convergence performance. This observation is further supported by the validation loss curve in Figure 2B, where the Swin-UNETR model shows no significant signs of overfitting and maintains a consistently low validation loss throughout the training process.

**Figure 2:**
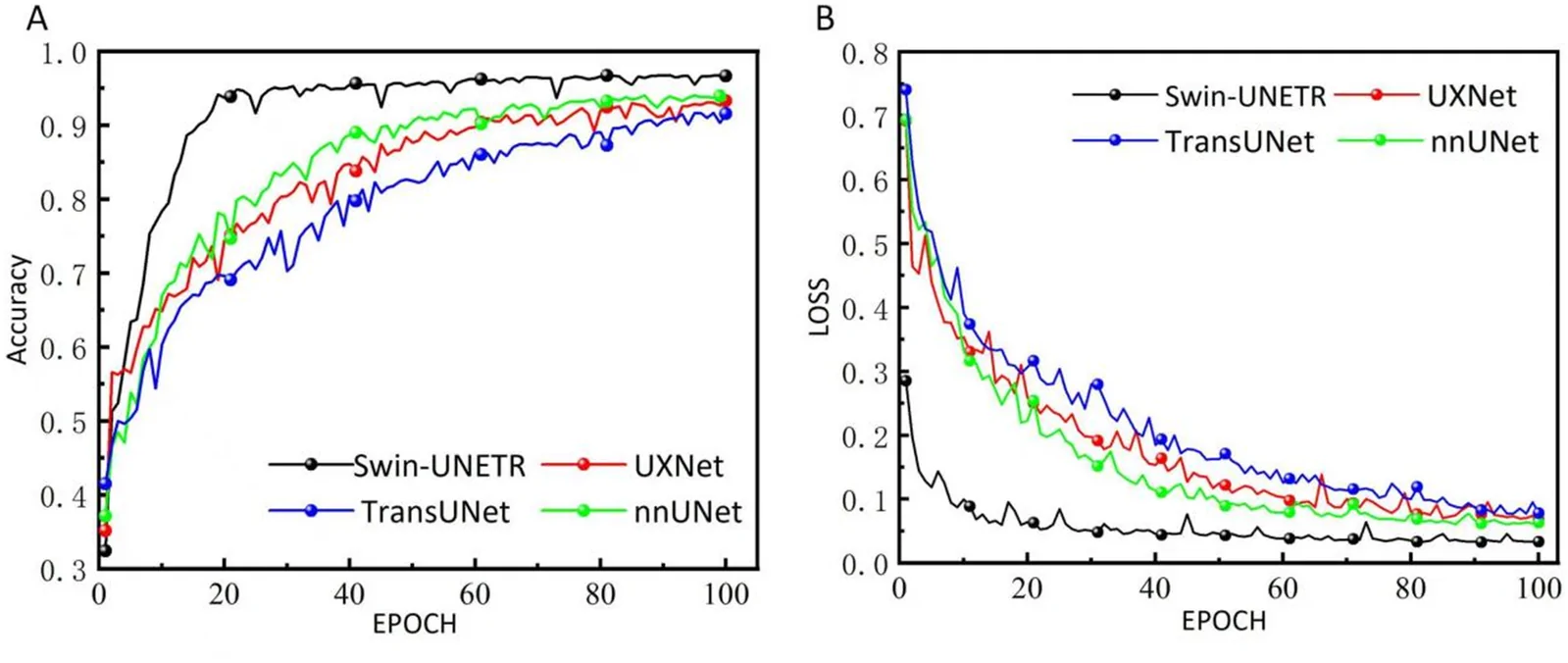
Model selection. **(A)** Performance curve; **(B)** Validation of loss curves.

### Comparison of model segmentation efficiency

The visual comparison of segmentation results from each model against senior observers manual annotations is presented in Figure 3A. The UXNet model exhibits a minor segmentation discontinuity at the bifurcation region between the tumor and the iliac artery, while the TransUNet model demonstrates insufficient accuracy in delineating the aneurysm neck, accompanied by localized over-segmentation. Furthermore, although the nnUNet model achieves generally favorable segmentation performance, it still suffers from partial loss of fine details at the junctions of small vessels. In contrast, the Swin-UNETR model shows excellent agreement with the gold standard in critical regions—including the aneurysm neck, iliac artery bifurcation, and vascular branch connections. Its segmentation contours are both continuous and precise, with no evident fractures or instances of over-segmentation and under-segmentation.

**Figure 3:**
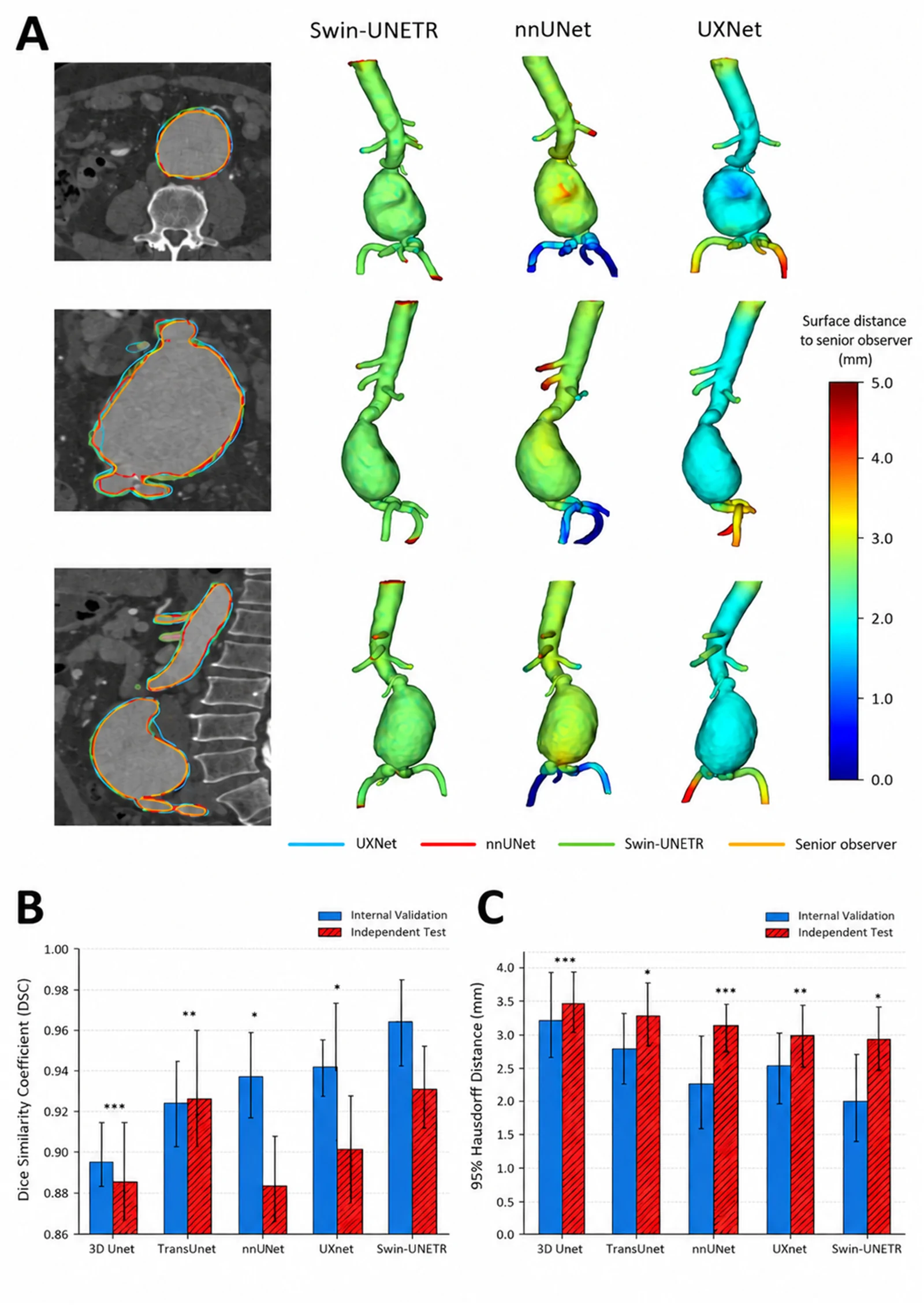
Models comparison and quantitative performance assessment of different models. **(A)** Comparison between different model algorithms and manual operation of experts; **(B)** Dice similarity coefficient (DSC) comparison results; **(C)** Comparison results of 95% Hausdorff distance (HD95).

To further quantify the segmentation performance, Figures 3B,C shows the DSC and HD95 of each model on the internal and independent test sets. On the internal validation set, the SWEin-UNETR achieved a DSC of 0.965 ± 0.025 and a HD95 of 2.01 ± 0.39 mm. Compared with nnUNet (DSC 0.937 ± 0.019, HD95 2.25 ± 0.64 mm), which had better performance in the comparison model, the DSC was further improved and the boundary error was further reduced. In the independent test set, Swin-UNETR still achieved the highest DSC (0.931 ± 0.021) and the lowest HD95 (2.93 ± 0.28 mm).

### Model performance

To further evaluate the effectiveness of the SSL framework, we performed patient-level paired statistical comparisons between the SSL model and the fully supervised Swin-UNETR model with the same backbone architecture, trained using the identical annotated dataset. On the internal validation set, the SSL model achieved a DSC of 0.965 ± 0.025, outperforming the fully supervised model's DSC of 0.934 ± 0.054 (*P* = 0.008). Similarly, the HD95 values were 2.01 ± 0.39 mm for the SSL model and 3.35 ± 0.28 mm for the fully supervised model, respectively (*P* = 0.029). This performance advantage was further evident on the more challenging independent test set: the DSC values were 0.931 ± 0.021 and 0.915 ± 0.039 (*P* = 0.035), while the corresponding HD95 values were 2.93 ± 0.28 mm and 3.57 ± 0.27 mm (*P* = 0.007), favoring the SSL approach.

### Measurement consistency

A comprehensive comparison of measurements across senior observers, internal cohorts, and independent test cohorts was conducted (Table 2). The results showed that there was no significant difference in the key morphological paramm among the three groups (all *P* > 0.05).

**Table 2:** Summary of the deviation and consistency analysis between the automatic measurement system and the manual gold standard.

Measurement parameter	Interobserver		Internal validation			Independent test		
	Bias	ICC	Bias	ICC	*P* value	Bias	ICC	*P* value
Maximum diameter of aneurysm, mm	−0.821 ± 3.37	0.961	0.198 ± 1.327	0.989	0.284	0.234 ± 1.428	0.994	0.312
Maximum diameter of iliac artery, mm	0.934 ± 3.58	0.943	0.215 ± 1.224	0.974	0.195	0.337 ± 1.128	0.980	0.086
Aneurysm neck diameter, mm	−0.912 ± 3.15	0.925	0.139 ± 1.632	0.995	0.541	0.194 ± 1.574	0.994	0.428
Length of aneurysm neck, mm	0.638 ± 2.37	0.942	0.248 ± 1.217	0.987	0.163	−0.328 ± 1.297	0.994	0.115
Proximal neck angle,°	1.685 ± 3.24	0.937	−0.249 ± 1.253	0.979	0.188	0.349 ± 1.36	0.976	0.124
Deflection angle of tumor cavit*y* axis (*β*),°	1.992 ± 2.29	0.929	0.288 ± 1.112	0.944	0.106	0.399 ± 1.98	0.973	0.185
Iliac artery bifurcation Angle,°	3.325 ± 5.10	0.889	−0.561 ± 2.48	0.901	0.237	0.674 ± 1.98	0.975	0.092

ICC, Intraclass correlation coefficient.

To further evaluate measurement reliability, we performed an in-depth assessment of agreement using ICC and Bland-Altman analysis (Figure 4). As shown in Figure 4A, the ICC analysis demonstrated generally good-to-excellent agreement between the automated and manual measurements across the evaluated morphological parameters. Bland-Altman analysis further confirmed the absence of significant systematic bias between the two measurement approaches (Figure 4B). The mean deviation of the maximum diameter of AAA was −0.4 mm, and the 95% limits of agreement (LoA) was [−2.8 mm, 2.0 mm]. For the iliac artery bifurcation Angle, the mean deviation was −0.4°, and the LoA was [−3.0°, 2.2°].

**Figure 4:**
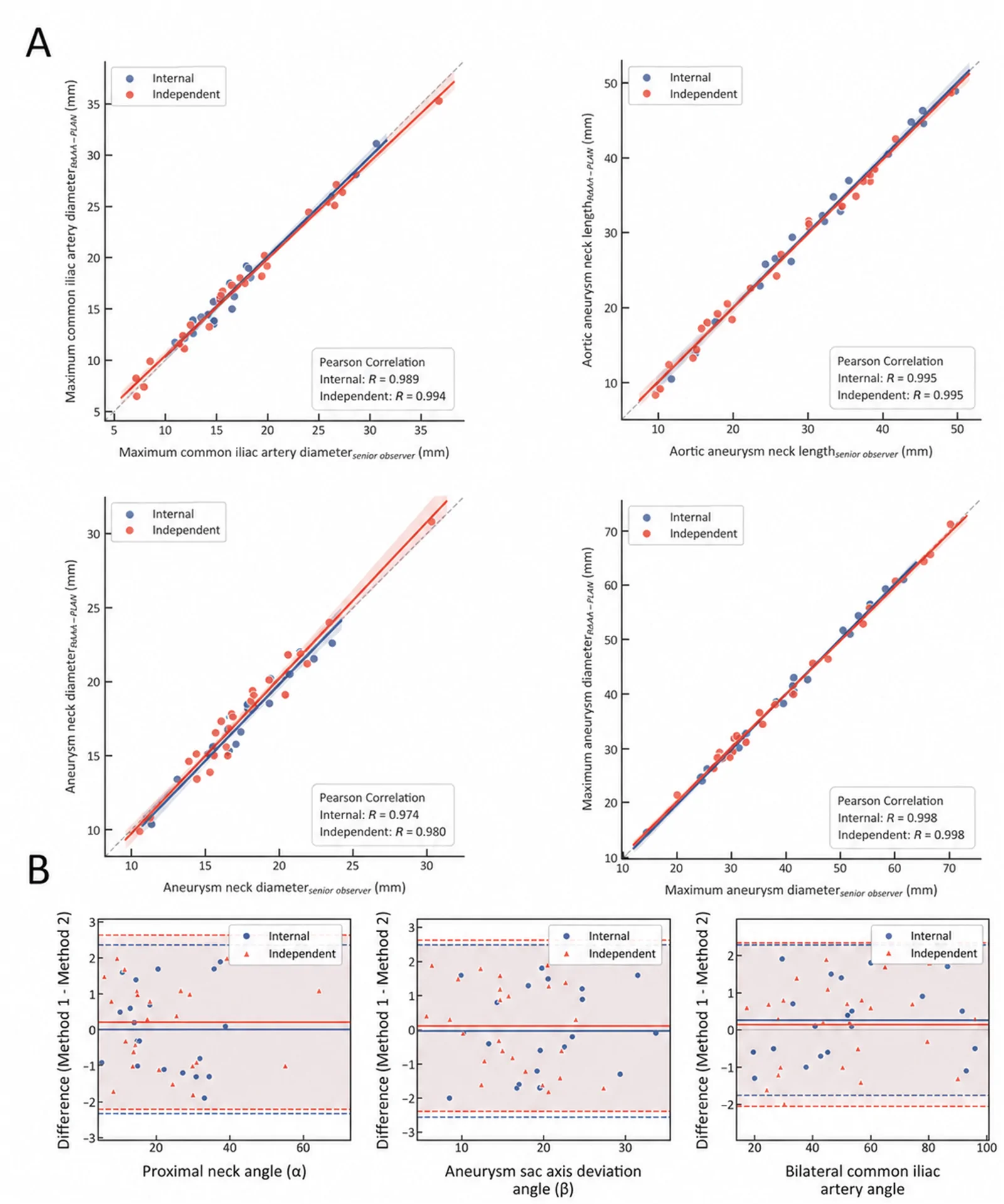
Model automated measurements. **(A)** ICC consistency analysis; **(B)** Bland-Altman consistency analysis.

### Efficiency assessment

In order to quantify the impact of the automated process on clinical workflow efficiency, this study systematically collected and compared key performance metrics between the automated process and senior observers manual evaluation, including the total time required for a single measurement cycle, the total distance traveled by the mouse cursor, and the total number of mouse clicks (Figure 5). The average time required to complete a full morphological assessment was significantly reduced from 53.08 ± 8.17 min with manual procedures to 4.18 ± 0.90 min using the automated approach (*P* < 0.001). Concurrently, the physical workload associated with user interaction was markedly decreased: the total length of the mouse trajectory was reduced from 91.62 ± 9.95 to 13.03 ± 3.12 m (*P* < 0.001), and the number of mouse clicks decreased from 672.62 ± 129.47 to 157.69 ± 0.90 times (*P* < 0.001).

**Figure 5:**
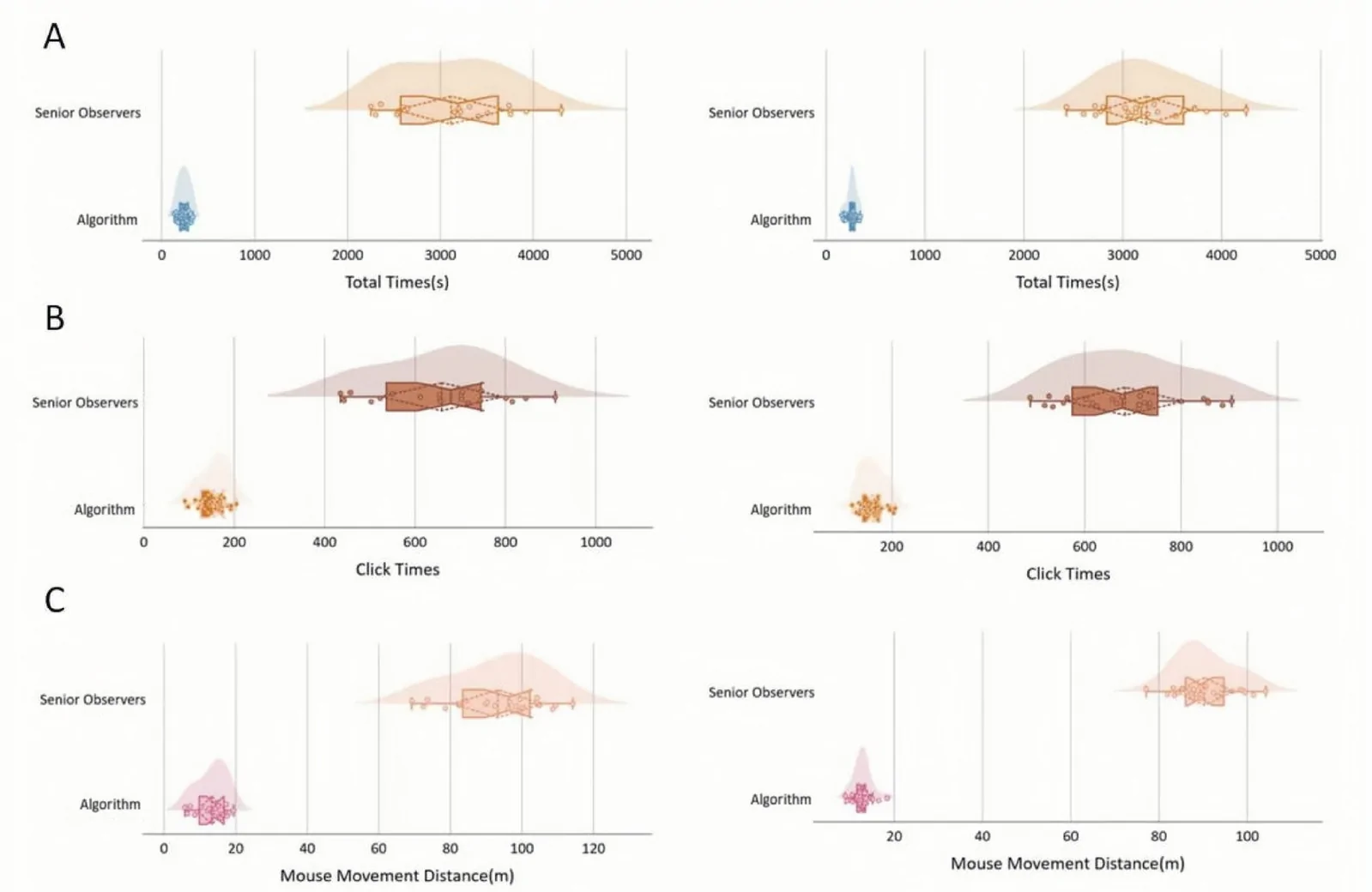
Model efficiency. **(A)** Total times; **(B)** Click times; **(C)** Mouse movement distance.

## Discussion

In this study, we successfully developed and validated a novel Swin-UNETR framework to implement a complete flow from automatic AAA segmentation to measurement of key anatomical features. The proposed SSL-based model achieves segmentation accuracy highly consistent with senior observers manual annotations, despite requiring only a limited amount of labeled training data. More importantly, the framework enables efficient extraction of multiple critical morphological paramm, with measurement results demonstrating strong agreement with those obtained manually by senior observers.

At present, preoperative planning for AAA imposes extremely high demands on imaging evaluation. The development of surgical strategies and detailed procedural planning heavily relies on accurate analysis of the three-dimensional morphological characteristics of the aneurysm, aneurysm neck, and iliac arteries derived from CTA scans (19). However, the prerequisite for obtaining these critical anatomical paramm is precise segmentation of the AAA. In current clinical practice, this process mainly relies on manual completion, which is not only time-consuming and laborious, but also easily affected by the operator's experience, leading to significant inter-senior observer differences (20). To address this challenge, fully supervised DL algorithms, such as U-Net, have demonstrated considerable potential for automated segmentation. Nevertheless, their performance typically depends on large-scale, high-quality datasets with pixel-level annotations (21). Consequently, developing a methodology that achieves high segmentation accuracy while minimizing labeling effort has become a crucial step toward facilitating the clinical translation of automated assessment tools. In this regard, SSL techniques are increasingly recognized as a key solution to overcome the data annotation challenges (22). SSL can substantially reduce reliance on manually labeled data while preserving the model's robustness and generalization capability (22), thereby paving the way for the deployment and widespread clinical adoption of such algorithms.

We have developed the first SSL-based automatic segmentation and measurement algorithm for AAA to date. The superior performance of the proposed algorithm is primarily attributed to the implementation of two key strategies, which effectively address the limitations of conventional approaches. First, the selection of the Swin-UNETR architecture plays a decisive role. Compared with prior studies (23), the incorporation of the shifted window self-attention mechanism from Transformer enables Swin-UNETR to capture global contextual information and long-range spatial dependencies in medical images with significantly enhanced computational efficiency. For AAA structures characterized by complex morphology and extensive spatial distribution, this capability effectively overcomes the inherent limitations of traditional convolutional neural NETworks in extracting long-distance features (24). Experimental results on the internal validation set demonstrate that the proposed model achieves outstanding segmentation performance, with an accuracy of 97.3%, indicating substantial performance advantages (25). In a previous study (25), even if a variety of advanced network architectures such as ResDenseUNet, INet, C-Net and their proposed optimization strategies are adopted, the accuracy rate is still only between 83.48% and 89.64%. In contrast, the proposed model achieves an improvement of approximately 7.6% in accuracy, strongly validating the superiority of the Swin-UNETR framework in segmenting complex vascular structures (25). Furthermore, the model attains a DSC of 0.965 ± 0.025, markedly outperforming the DSC intervals of 0.89 ± 0.04 and 0.95 ± 0.01 reported by Roby and Caradu et al., respectively, based on U-NET–derived models (20, 26). Notably, the model's HD95 value is only 2.01 ± 0.39 mm, substantially lower than the 4.61 ± 7.26 mm reported by Caradu et al., further confirming its high geometric fidelity in delineating the fine boundaries between AAA and surrounding soft tissues (26).

Secondly, the incorporation of the SSL strategy may reduce reliance on large-scale labeled datasets, potentially mitigate overfitting issue during training under small-sample labeling conditions, and contribute to improved model generalization (25). On the independent test dataset, the proposed model maintained strong performance (DSC: 0.931 ± 0.021, HD95: 2.93 ± 0.28 mm), which clearly demonstrates its robustness. Together with the controlled comparison against the fully supervised Swin-UNETR model, these findings suggest that incorporating the SSL strategy may provide additional performance benefits when labeled AAA imaging data are limited.

Furthermore, the high-precision segmentation achieved by the proposed model represents not merely a technical advancement, but a robust foundation for delivering tangible clinical value. The automated workflow enabled a substantial improvement in the efficiency of clinical assessments: the average measurement time was significantly reduced from 53.08 ± 8.17 min to 4.18 ± 0.90 min; the total length of the mouse trajectory was shortened from 91.62 ± 9.95 to 13.03 ± 3.12 m; and the number of mouse clicks decreased markedly from 672.62 ± 129.47 to 157.69 ± 0.90 times. These significant gains in efficiency are crucial for streamlining clinical workflows, particularly in time-sensitive scenarios such as the assessment of acute rupture risk (27). At the same time, the algorithm showed a high consistency with the standardized measurement results. Consistent with the favorable agreement reported by Brutti et al., the present model also demonstrated high measurement consistency across the evaluated cohorts (20). The automated process significantly reduces both inter-operator and intra-operator variability inherent in traditional manual measurements, which represents a critical factor influencing the accuracy of preoperative assessment of AAA and the determination of optimal intervention timing (28). By delivering highly reproducible and objective quantitative data, the framework developed in this study offers reliable support for optimizing clinical decision-making, thereby addressing the increasing demand for interpretable and standardized AAA assessment in contemporary clinical practice.

In addition, although the model demonstrates considerable application potential, it is imperative to critically assess its limitations with rigorous scientific scrutiny to provide clear guidance for future research. First, the data utilized in this study were collected from a single center, which ensures homogeneity and consistency in the data acquisition process. Furthermore, the overall sample size of this study is relatively limited, with the independent test set in particular being small in size. The single-center design and constrained sample size have, to a certain extent, restricted the generalizability of the model—most notably, as the model has not yet undergone prospective validation across multicenter settings, diverse imaging platforms, or varied scanning protocols. Second, rare cases with extremely complex morphology were not included in this study, such as AAA patients with severely distorted necks, giant mural thrombi, or complicated iliac aneurysms. Consequently, the robustness of the current model in managing such challenging clinical scenarios requires further evaluation and refinement through more comprehensive independent test using diverse datasets. Finally, the SSL workflow inherently operates as a sequential pipeline, wherein the accuracy of the final quantitative measurements is critically dependent on the precision of upstream processes, including segmentation and centerline extraction. Although the present model has achieved remarkably low error rates, any residual segmentation inaccuracies may propagate progressively through subsequent stages, potentially compromising the reliability of downstream analyses. In addition, although the controlled comparison with a fully supervised Swin-UNETR model suggests a potential benefit of the SSL strategy, the present study did not perform exhaustive ablation experiments across different backbone architectures or individual SSL components; therefore, their respective contributions to the observed performance improvement cannot be fully disentangled.

## Conclusion

In conclusion, the SSL algorithm integrated with the Swin-UNETR architecture proposed in this study offers robust technical support for optimizing clinical decision-making and enhancing the prognosis of patients with AAA, by enabling efficient and accurate automatic segmentation as well as quantitative assessment of AAA.

## Data Availability

The raw data supporting the conclusions of this article will be made available by the authors, without undue reservation.
